# Ca^2+^/Calmodulin-Dependent Protein Kinase II Regulation by Inhibitor of Receptor Interacting Protein Kinase 3 Alleviates Necroptosis in Glycation End Products-Induced Cardiomyocytes Injury

**DOI:** 10.3390/ijms23136988

**Published:** 2022-06-23

**Authors:** Yuyun Hua, Jianan Qian, Ji Cao, Xue Wang, Wei Zhang, Jingjing Zhang

**Affiliations:** 1 School of Pharmacy, Nantong University, Nantong 226001, China; huayuyun@outlook.com (Y.H.); qjn29103@163.com (J.Q.); caojie@stmail.ntu.edu.cn (J.C.); w18862936086@163.com (X.W.); 2 School of Medicine, Nantong University, Nantong 226001, China

**Keywords:** advanced glycation end products, cardiomyocytes, necroptosis, receptor interacting protein kinase 3, calcium/calmodulin-dependent protein kinase II, oxidative stress

## Abstract

Necroptosisis a regulatory programmed form of necrosis. Receptor interacting protein kinase 3 (RIPK3) is a robust indicator of necroptosis. RIPK3 mediates myocardial necroptosis through activation of calcium/calmodulin-dependent protein kinase II (CaMKII) in cardiac ischemia-reperfusion (I/R) injury and heart failure. However, the exact mechanism of RIPK3 in advanced glycation end products (AGEs)-induced cardiomyocytes necroptosis is not clear. In this study, cardiomyocytes were subjected to AGEs stimulation for 24 h. RIPK3 expression, CaMKII expression, and necroptosis were determined in cardiomyocytes after AGEs stimulation. Then, cardiomyocytes were transfected with RIPK3 siRNA to downregulate RIPK3 followed by AGEs stimulation for 24 h. CaMKIIδ alternative splicing, CaMKII activity, oxidative stress, necroptosis, and cell damage were detected again. Next, cardiomyocytes were pretreated with GSK′872, a specific RIPK3 inhibitor to assess whether it could protect cardiomyocytes against AGEs stimulation. We found that AGEs increased the expression of RIPK3, aggravated the disorder of CaMKII δ alternative splicing, promoted CaMKII activation, enhanced oxidative stress, induced necroptosis, and damaged cardiomyocytes. RIPK3 downregulation or RIPK3 inhibitor GSK′872 corrected CaMKIIδ alternative splicing disorder, inhibited CaMKII activation, reduced oxidative stress, attenuated necroptosis, and improved cell damage in cardiomyocytes.

## 1. Introduction

Diabetic cardiomyopathy (DCM) is defined by the existence of myocardial structural abnormality and dysfunction in diabetes mellitus (DM) without coronary artery disease, valve disease, hypertension, dyslipidemia, and other cardiovascular risk factors. The incidence rate of diabetes cardiomyopathy in patients with diabetes was 16.9%, and the mortality and disability rates were about 18% and 22%, respectively [1,2]. The early stage of DCM manifests as recessive subclinical cardiac abnormalities, such as fibrosis, myocardial hypertrophy and diastolic dysfunction. Then, it develops into systolic dysfunction, eventually leading to heart failure [3]. Clinical trials have shown that the incidence of heart failure in DM is between 19% and 26%, which is much higher than that in non-diabetic patients [4,5]. The data suggests that elevated blood glucose is an important contributor to heart failure in DM. The specific pathogenesis of DCM is still unclear. It is an urgent scientific problem to clarify the pathogenesis of DCM and seek a reliable drug target for the prevention and treatment of DCM.

Long-term hyperglycemia in DM induces a non-enzymatic reaction between glucose and proteins, lipids or nucleic acids, promoting advanced glycation end products (AGEs) production [6]. Glycosylation leads to enzyme disfunction, protein cross-linking or aggregation, cell function and signal pathway disorder, and tissue damage [7]. AGEs accumulation plays an important role in numerous diseases, such as diabetes, immune inflammation, cardiovascular, and neurodegenerative diseases [8,9,10]. The patients with cardiometabolic diseases reduced the intake of AGEs, significantly decreased the serum AGEs levels, and improved the imbalance of redox regulation [11,12]. In addition, reducing the intake of exogenous AGEs in type 2 diabetes or obesity can also effectively increase insulin sensitivity, inhibit inflammation, and improve mitochondrial function [13,14]. The above studies suggest that AGEs play a key role in the occurrence and development of DCM.

Cell death is a fundamental phenomenon in development with distinct manifestations, including necrosis, apoptosis, and autophagy [15]. Apoptosis is a strictly controlled process, while necrosis is considered to be a passive and sporadic type of cell death. Another type of cell death is called necroptosis [16]. Necroptosis is closely related to embryonic development [17], inflammation [18], neurodegeneration [19], tumors [20], cardiovascular disease [21], and other physiological and pathological processes. Receptor interacting proteins kinase 3 (RIPK3) is a central indicator of necroptosis [22]. RIPK3 drives the downstream molecule calcium/calmodulin-dependent protein kinase II (CaMKII), causing myocardial necroptosis in cardiac ischemia/reperfusion (I/R) injury and heart failure [23]. In addition, GSK′872, a small molecule RIPK3 inhibitor, blocked TNF-α induced necroptosis in human HT-29 cells in a concentration-dependent manner [24]. However, the role and mechanism of RIPK3 in AGEs-induced cardiomyocyte necroptosis is still unclear.

CaMKII is encoded by different genes to form four subtypes, namely α, β, γ, and δ, which show different but partially overlapping expression patterns [25,26]. CaMKII δ alternative splicing occurs between exons 13–17 and 20–22, resulting in at least 11 splice variants [27]. CaMKII δA, CaMKII δB, and CaMKII δC are major splice variants in the heart. CaMKII δA is mainly located in T-tubules, muscle membranes, and nuclear membranes, and are related to chronic heart failure [28]. CaMKII δB contains a nuclear localization sequence, mainly located in the nucleus, regulating transcriptional functions [29]. CaMKII δC is mainly located in the cytoplasm and affects cardiac excitation–contraction coupling [30]. Overall activation of CaMKII was upregulated 3-fold, and CaMKII δ expression was increased 2-fold in heart failure [31]. However, it is not clear whether AGEs-induced necroptosis depends on CaMKII activation mediated by RIPK3in cardiomyocytes.

In summary, this study first clarified the changes in RIPK3, CaMKII, and necroptosis in AGEs-induced cardiomyocyte injury. Then, we explored whether downregulation of RIPK3 could regulate CaMKII δ altered splicing and CaMKII activity, inhibit oxidative stress, alleviate necroptosis, and reduce cell damage through RIPK3 siRNA interference or RIPK3 inhibitor GSK′872. The study is conducive for clarifying the new pathogenesis of DCM and provides a new scientific basis for clinical prevention and treatment of DCM and related drug transformation.

## 2. Results

### 2.1. AGEs-Damaged Cardiomyocytes

**Figure 1:**
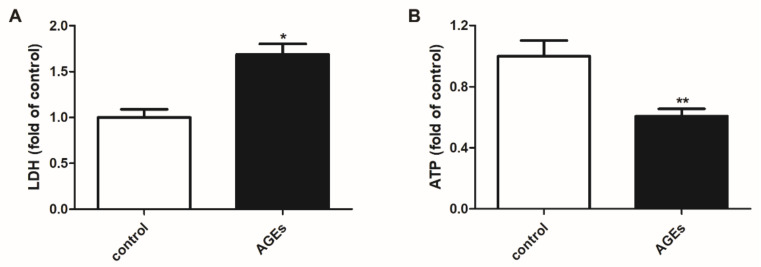
AGEs damaged cardiomyocytes. Cardiomyocytes were stimulated with AGEs (200 μg/mL) for 24 h. (**A**) LDH release into the culture medium was detected by LDH Cytotoxicity Assay Kit. (**B**) Cellular ATP level was detected by Cell Titer-Lumi™ Luminescent Cell Viability Assay Kit. Plots represent the mean ± SEM; n = 6. Statistical significance: * *p* < 0.05, ** *p* < 0.01 compared with control.

A variety of enzymes in the cytoplasm are released into the culture medium due to cell membrane destruction. Among them, the enzyme activity of LDH is relatively stable. Therefore, the LDH release is considered as an important indicator for the integrity of the cell membrane. ATP is a common energy carrier and is closely related to important physiological and pathological processes. Normally, a drop in ATP levels indicates impaired cell function. After AGEs stimulation for 24 h, LDH release into the culture medium and cellular ATP level was detected to evaluate cardiomyocyte damage. The study found that AGEs increased LDH release, but decreased ATP level (Figure 1A,B).

### 2.2. AGEs Induced Necroptosis in Cardiomyocytes

**Figure 2:**
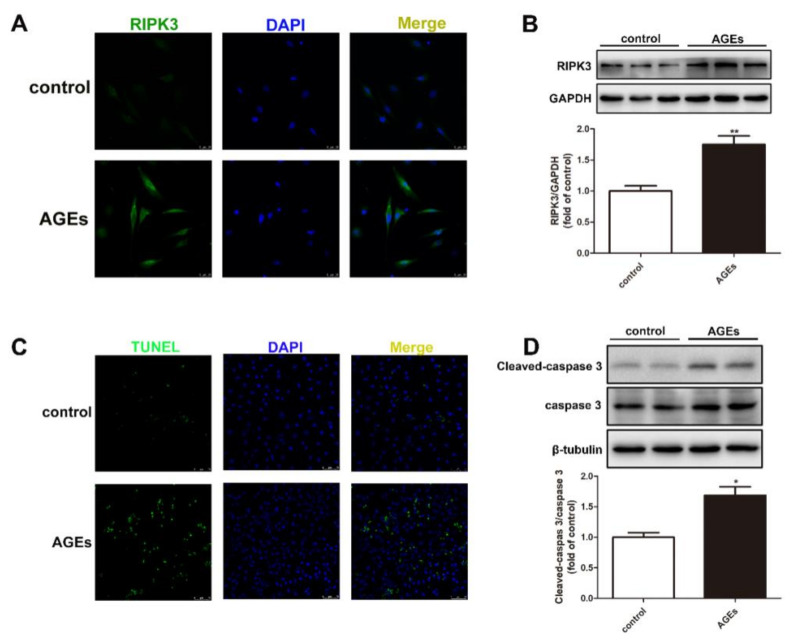
AGEs induced necroptosis in cardiomyocytes. Cardiomyocytes were stimulated with AGEs (200 μg/mL) for 24 h. (**A**) RIPK3 was immunofluorescence stained by Alexa Fluor 488 (Green)-conjugated IgG. The nuclei were stained by DAPI (Blue). Bar = 25 μm. (**B**) The expression of RIPK3 was detected by Western blot in cardiomyocytes. GAPDH was used as a loading control. (**C**) The apoptosis of cardiomyocytes was detected by One Step TUNEL Apoptosis Assay Kit. Bar = 75 μm. (**D**) The expression of caspase 3 and Cleaved-caspase 3 was detected by Western blot in cardiomyocytes. β-tubulin was used as a loading control. Plots represent the mean ± SEM; n = 6. Statistical significance: * *p* < 0.05, ** *p* < 0.01 compared with control.

After AGEs stimulation for 24 h, the expression ofRIPK3 in the cardiomyocytes was detected by immunofluorescence and Western blot. We found that AGEs increased the expression of RIPK3 (Figure 2A,B).

Apoptosis is also an important manifestation of necroptosis. After AGEs stimulation for 24 h, the expression of Cleaved-caspase 3 and caspase 3 were detected by Western blot and the apoptosis of cardiomyocytes was detected by TUNEL staining. The results showed that AGEs increased the expression of Cleaved-caspase 3, enhanced the ratio of TUNEL positive cardiomyocytes, indicating that the level of cardiomyocyte apoptosis increased (Figure 2C,D). The above data showed that AGEs aggravated the necroptosis in cardiomyocytes.

### 2.3. AGEs Aggravated the Disorder of CaMKIIδ Alternative Splicing in Cardiomyocytes

**Figure 3:**
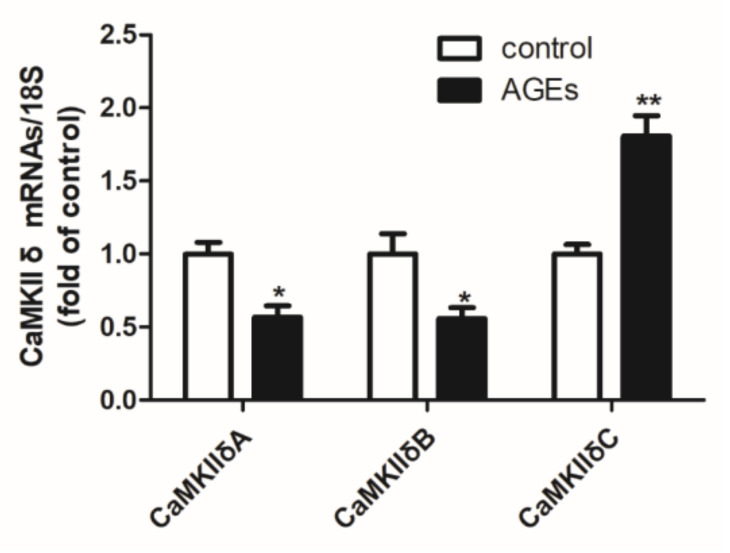
AGEs aggravated the disorder of CaMKIIδ alternative splicing in cardiomyocytes. Cardiomyocytes were stimulated with AGEs (200 μg/mL) for 24 h. The mRNA expression of CaMKIIδA, CaMKIIδB, and CaMKIIδC was detected by quantitative real-time PCR in cardiomyocytes. 18S was used as a loading control. Plots represent the mean ± SEM; n = 6. Statistical significance: * *p* < 0.05, ** *p* < 0.01 compared with control.

Since there is no specific antibody against CaMKII δ variants, quantitative real-time PCR was used to detect the gene expression of CaMKIIδA, CaMKIIδB, and CaMKIIδC mRNA in cardiomyocytes after stimulation with 200 μg/mL AGEs for 24 h. The study found that AGEs decreased the expression of CaMKII δA and CaMKII δB, but increased the expression of CaMKII δC in cardiomyocytes, suggesting CaMKIIδ alternative splicing was disordered after AGEs stimulation (Figure 3).

### 2.4. AGEs Promoted CaMKII Activation in Cardiomyocytes

**Figure 4:**
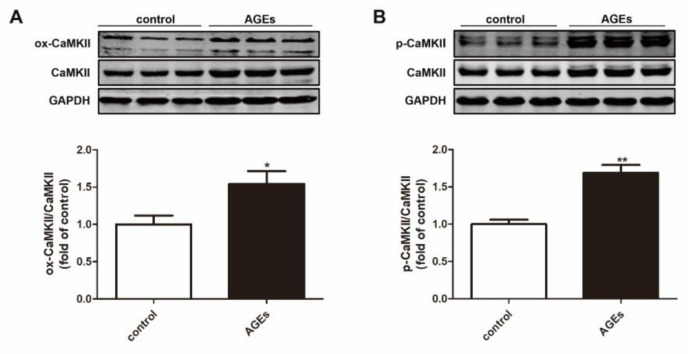
AGEs promoted CaMKII activation in cardiomyocytes. Cardiomyocytes were stimulated with AGEs (200 μg/mL) for 24 h. (**A**) The expression of ox-CaMKII and CaMKII was detected by Western blot in cardiomyocytes. GAPDH was used as a loading control. (**B**) The expression of p-CaMKII and CaMKII was detected by Western blot in cardiomyocytes. GAPDH was used as a loading control. Plots represent the mean ± SEM; n = 6. Statistical significance: * *p* < 0.05, ** *p* < 0.01 compared with control.

Previous studies have found that CaMKII can be activated through oxidation or phosphorylation. After AGEs stimulation for 24 h, the expression of ox-CaMKII and p-CaMKII were detected by Western blot. The results showed that AGEs increased the expression of ox-CaMKII and p-CaMKII (Figure 4).

### 2.5. siRNA Transfection Downregulated RIPK3 Expression in Cardiomyocytes

**Figure 5:**
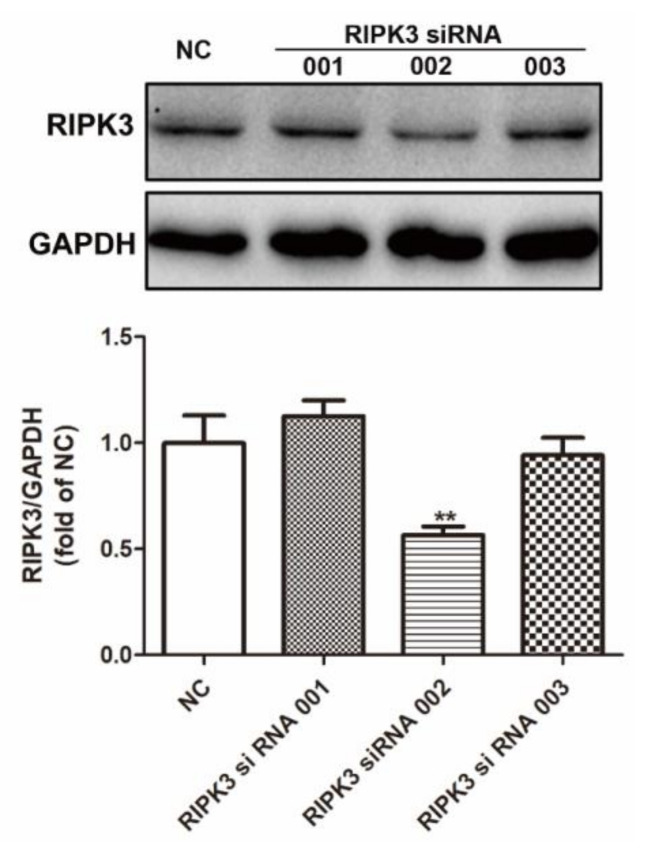
siRNA transfection downregulated RIPK3 expression in cardiomyocytes. Cardiomyocytes were transfected with NC siRNA or RIPK3 siRNA (001, 002, 003) for 4 h and cultured in DME/F-12 medium containing 10% FBS for 24 h. The expression of RIPK3 was detected by Western blot in cardiomyocytes. GAPDH was used as a loading control. Plots represent the mean ± SEM; n = 6. Statistical significance: ** *p* < 0.01 compared with NC.

It was unclear whether inhibiting RIPK3 expression had a protective effect on cardiomyocytes damaged by AGEs. Did RIPK3 act as an upstream of CaMKII to regulateCaMKII alternative splicing in cardiomyocytes? Could correcting the variants inhibit the necroptosis of cardiomyocytes AGEs-induced by disorder of CaMKII? Therefore, RIPK3 expression was downregulated with RIPK3 siRNA transfection. Then, cell damage, necroptosis, CaMKII δ alternative splicing, and CaMKII activation were detected again. The transfection efficiency and effect of RIPK3 siRNA were detected by Western blot. RIPK3 siRNA 002 significantly reduced the expression of RIPK3 (Figure 5). Therefore, our follow-up experiments were transfected with RIPK3 siRNA 002 to down regulate the expression of RIPK3 in cardiomyocytes.

### 2.6. Downregulation of RIPK3 Attenuated Cell Damage in Cardiomyocytes

**Figure 6:**
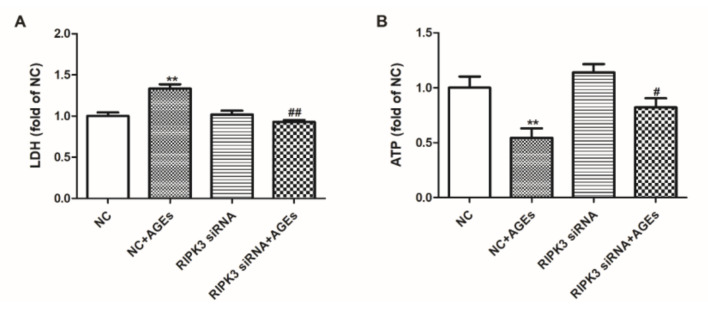
Downregulation of RIPK3 attenuated cell damage in cardiomyocytes. Cardiomyocytes were transfected with RIPK3 siRNA for 4 h followed by 200 μg/mL AGEs stimulation for 24 h. (**A**) LDH release into the culture medium was detected by LDH Cytotoxicity Assay Kit. (**B**) Cellular ATP level was detected by CellTiter-Lumi™ Luminescent Cell Viability Assay Kit. Plots represent the mean ± SEM; n = 6. Statistical significance: ** *p* < 0.01 compared with NC; ^#^ *p* < 0.05, ^##^ *p* < 0.01 compared with NC+AGEs.

The cardiomyocytes were transfected with RIPK3 siRNA for 4 h followed by AGEs stimulation for 24 h. LDH released into the culture medium and cellular ATP level were detected to evaluate the cell damage. The results revealed that downregulation of RIPK3 reduced LDH release, improved ATP level, and attenuated cell damage in cardiomyocytes (Figure 6A,B).

### 2.7. Downregulation of RIPK3 Inhibited Necroptosisin Cardiomyocytes

**Figure 7:**
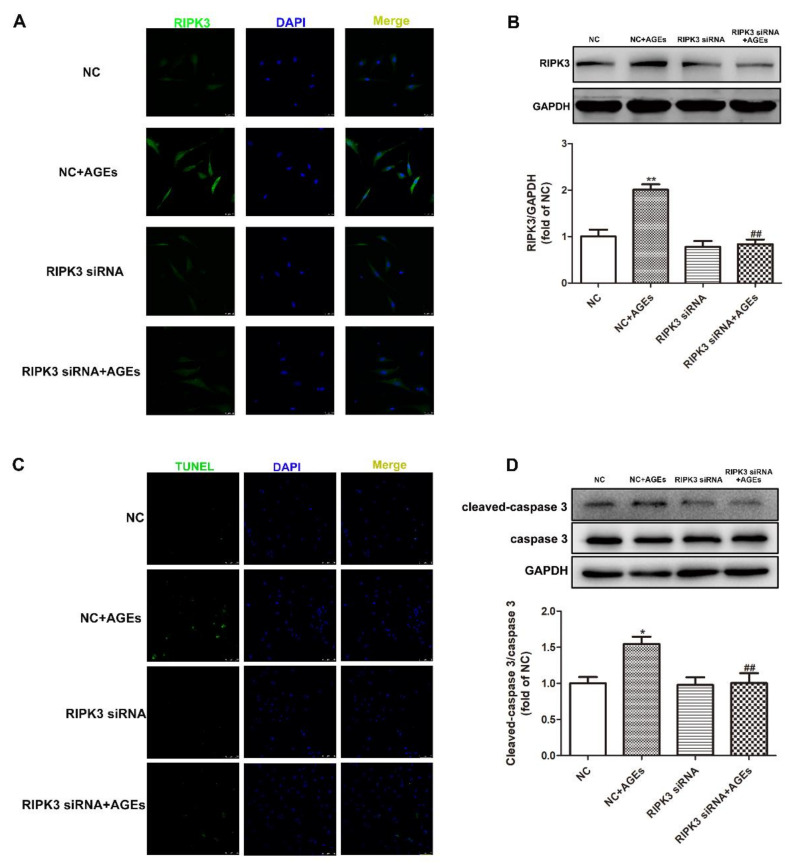
Down regulation of RIPK3 inhibited necroptosis in cardiomyocytes. Cardiomyocytes were transfected with RIPK3 siRNA for 4 h followed by 200 μg/mL AGEs stimulation for 24 h. (**A**) RIPK3 was immunofluorescence stained by Alexa Fluor 488 (Green)-conjugated IgG. The nuclei were stained by DAPI (Blue). Bar = 25 μm. (**B**) The expression of RIPK3was detected by Western blot in cardiomyocytes. GAPDH was used as a loading control. (**C**) The apoptosis of cardiomyocytes was detected by One Step TUNEL Apoptosis Assay Kit. Bar = 75 μm. (**D**) The expression of caspase 3 and Cleaved-caspase 3 was detected by Western blot in cardiomyocytes. GAPDH was used as a loading control. Plots represent the mean ± SEM; n = 6. Statistical significance: * *p* < 0.05, ** *p* < 0.01 compared with NC; ^##^ *p* < 0.01 compared with NC+AGEs.

The cardiomyocytes were transfected with RIPK3 siRNA for 4 h followed by AGEs stimulation for 24 h. The expression of RIPK3, Cleaved-caspase 3, caspase 3, and the apoptosis of cardiomyocytes was detected to explore whether downregulation of RIPK3 could inhibit AGEs-induced necroptosis. The results showed that downregulation of RIPK3 decreased the expression of RIPK3 (Figure 7A,B) and Cleaved-caspase 3 (Figure 7D), reduced the ratio of TUNEL positive cardiomyocytes (Figure 7C), and inhibited the necroptosis.

### 2.8. Downregulation of RIPK3 Ameliorated the Alternative Splicing Disorder of CaMKIIδ in Cardiomyocytes

**Figure 8:**
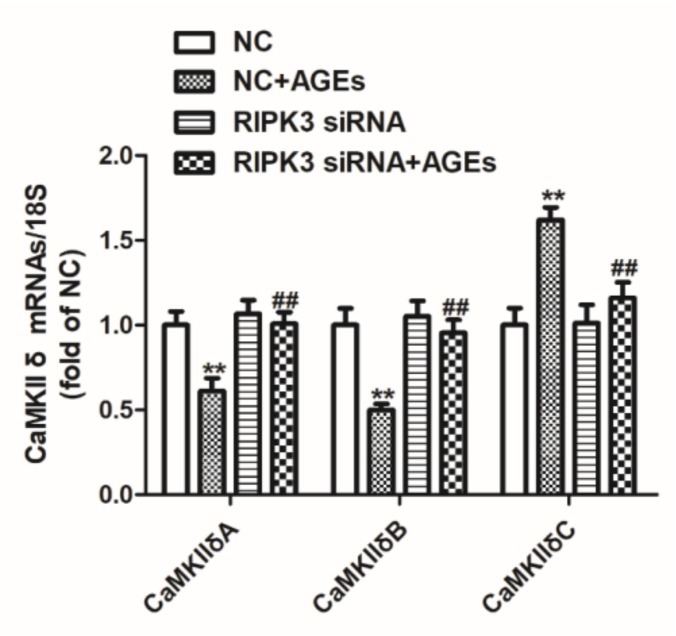
Down regulation of RIPK3 ameliorated the alternative splicing disorder of CaMKIIδ in cardiomyocytes. Cardiomyocytes were transfected with RIPK3 siRNA for 4 h followed by 200 μg/mL AGEs stimulation for 24 h. The mRNA expression of CaMKIIδA, CaMKIIδB, and CaMKIIδC was detected by quantitative real-time PCR in cardiomyocytes. 18S was used as a loading control. Plots represent the mean ± SEM; n = 6. Statistical significance: ** *p* < 0.01 compared with NC; ^##^ *p* < 0.01 compared with NC+AGEs.

The gene expression of CaMKII variants was detected by real-time PCR after transfection of RIPK3 siRNA for 4 h followed by AGEs stimulation for 24 h. The study found that downregulation of RIPK3 significantly increased the expression of CaMKIIδA and CaMKIIδB, and decreased the expression of CaMKIIδC, indicating that downregulation of RIPK3 corrected AGEs-induced CaMKIIδ alternative splicing disorder (Figure 8).

### 2.9. Downregulation of RIPK3 Inhibited CaMKII Activation in Cardiomyocytes

**Figure 9:**
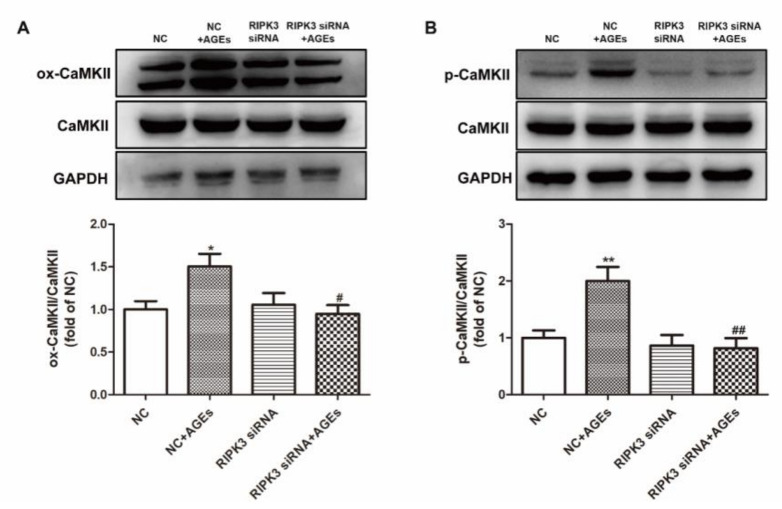
Downregulation of RIPK3 inhibited CaMKII activation in cardiomyocytes. Cardiomyocytes were transfected with RIPK3 siRNA for 4 h followed by 200 μg/mL AGEs stimulation for 24 h. (**A**) The expression of ox-CaMKII and CaMKII was detected by Western blot in cardiomyocytes. GAPDH was used as a loading control. (**B**) The expression of p-CaMKII and CaMKII was detected by Western blot in cardiomyocytes. GAPDH was used as a loading control. Plots represent the mean ± SEM; n = 6. Statistical significance: * *p* < 0.05, ** *p* < 0.01 compared with NC; ^#^ *p* < 0.05, ^##^ *p* < 0.01 compared with NC+AGEs.

The expression of ox-CaMKII and p-CaMKII was detected after transfection of RIPK3 siRNA for 4 h followed by AGEs stimulation for 24 h. The study found that downregulation of RIPK3 decreased the expression of ox-CaMKII and p-CaMKII (Figure 9), indicating that down regulating RIPK3 inhibited CaMKII activation induced by AGEs.

### 2.10. Downregulation of RIPK3 Reduced Oxidative Stress in Cardiomyocytes

**Figure 10:**
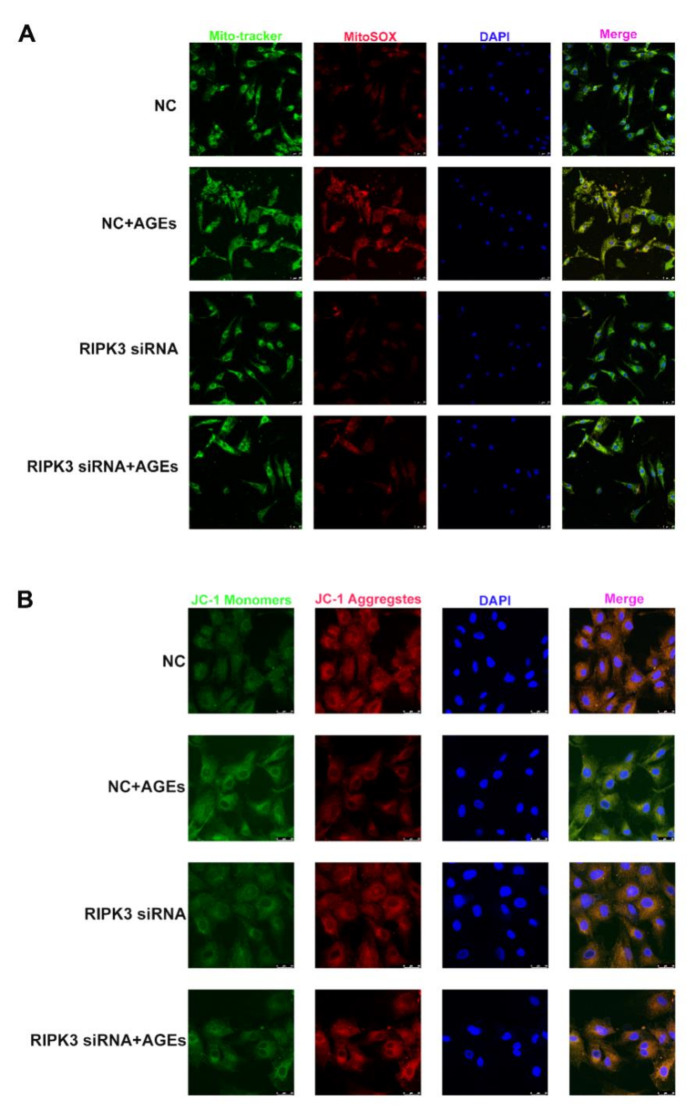
Down regulation of RIPK3 reduced oxidative stress in cardiomyocytes. Cardiomyocytes were transfected with RIPK3 siRNA for 4 h followed by 200 μg/mL AGEs stimulation for 24 h. (**A**) The generation of superoxide anion reactive oxygen species in mitochondria was detected by MitoSOX (Red) staining. Mito-Tracker (Green) co-localized mitochondria. The nuclei were stained by DAPI (Blue). Bar = 25 μm. (**B**) Mitochondrial membrane potential was detected by JC-1staining. When it is high, JC-1 aggregates in the matrix (Red). When it is low, JC-1 is mainly monomers (Green). The nuclei were stained by DAPI (Blue). Bar = 25 μm.

The intracellular oxidative stress increase causes cell and tissue damage. Superoxide anion is the main reactive oxygen species in the mitochondria, and its level can be detected by MitoSOX staining. The decrease in mitochondrial membrane potential induces excessive production of ROS, which aggravates the impairment of mitochondrial function. The level of mitochondrial membrane potential can be detected by JC-1 staining. The study found that AGEs increased mitochondrial ROS (Figure 10A), decreased mitochondrial membrane potential (Figure 10B), and enhanced oxidative stress; downregulation of RIPK3 reduced the production of mitochondrial ROS (Figure 10A) and improved mitochondrial membrane potential (Figure 10B) to attenuate oxidative stress.

### 2.11. GSK′872 Pretreatment Inhibited Necroptosis in Cardiomyocytes

**Figure 11:**
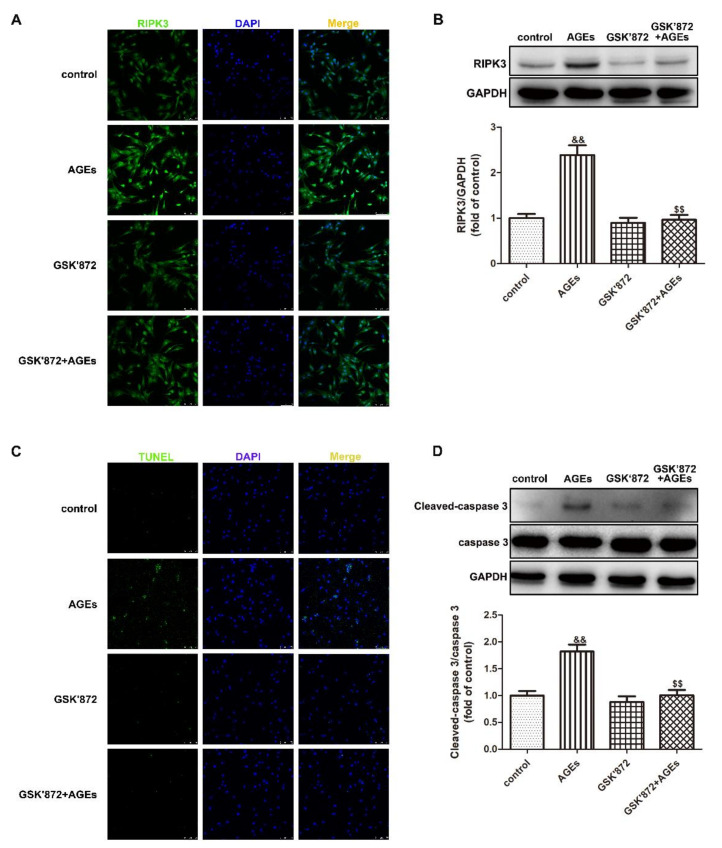
GSK′872 pretreatment inhibited necroptosis in cardiomyocytes. Cardiomyocytes were pretreated with 10 μM GSK′872 for 4 h followed by 200 μg/mL AGEs stimulation for 24 h. (**A**) RIPK3 was immunofluorescence stained by Alexa Fluor 488 (Green)-conjugated IgG. The nuclei were stained by DAPI (Blue). Bar = 75 μm. (**B**) The expression of RIPK3was detected by Western blot in cardiomyocytes. GAPDH was used as a loading control. (**C**) The apoptosis of cardiomyocytes was detected by One Step TUNEL Apoptosis Assay Kit. Bar = 75 μm. (**D**) The expression of caspase 3 and Cleaved-caspase 3 was detected by Western blot in cardiomyocytes. GAPDH was used as a loading control. Plots represent the mean ± SEM; n = 6. Statistical significance: ^&&^ *p* < 0.01 compared with control; ^$$^ *p* < 0.01 compared with AGEs.

The above results confirmed that downregulation of RIPK3 alleviated AGEs-induced necroptosis of cardiomyocytes and had a protective effect on cardiomyocytes. Next, we suppressed the expression of RIPK3 by pharmacological means, and provided a basis for finding a new drug that antagonizes diabetic myocardial damage. GSK′872 is a small molecule specific RIPK3 inhibitor. Cardiomyocytes were pretreated with 10 μM GSK′872 for 4 h followed by 200 μg/mL AGEs stimulation for 24 h. The expression of RIPK3, Cleaved-caspase 3, caspase 3, and the apoptosis of cardiomyocytes were assessed to explore whether GSK′872 alleviated the necroptosis induced by AGEs. The results showed that GSK′872 pretreatment significantly inhibited the expression of RIPK3 (Figure 11A,B) and Cleaved-caspase 3 (Figure 11D), reduced the ratio of TUNEL positive cardiomyocytes (Figure 11C), indicating that RIPK3 inhibitor GSK′872 attenuated necroptosis of cardiomyocytes induced by AGEs.

### 2.12. GSK′872 Pretreatment Ameliorated the Alternative Splicing Disorder of CaMKIIδ in Cardiomyocytes

**Figure 12:**
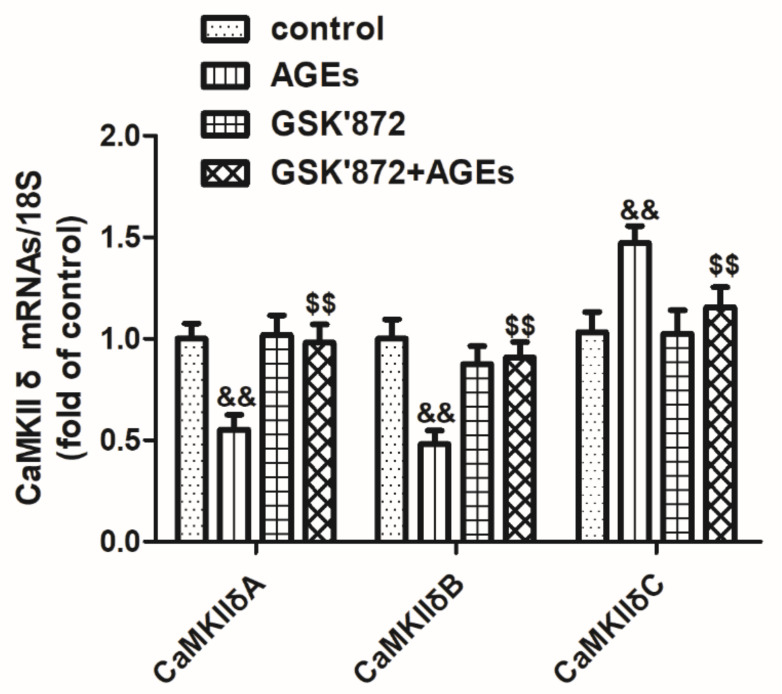
GSK′872 pretreatment ameliorated the alternative splicing disorder of CaMKII δ in cardiomyocytes. Cardiomyocytes were pretreated with 10 μM GSK′872 for 4 h followed by 200 μg/mL AGEs stimulation for 24 h. The mRNA expression of CaMKII δA, CaMKII δB, and CaMKII δC was detected by quantitative real-time PCR in cardiomyocytes. 18S was used as a loading control. Plots represent the mean ± SEM; n = 6. Statistical significance: ^&&^ *p* < 0.01 compared with control; ^$$^ *p* < 0.01 compared with AGEs.

Cardiomyocytes were pretreated with GSK′872 for 4 h followed by 200 μg/mL AGEs stimulation for 24 h. Use 10 μM GSK′872 mRNA expression of CaMKII δA, CaMKII δB, and CaMKII δC were detected by real-time PCR. The study found that GSK′872 pretreatment increased the expression of CaMKII δA and CaMKII δB, but decreased the expression of CaMKII δC, suggesting that GSK′872 corrected the AGEs-induced CaMKII δ alternative splicing disorder in cardiomyocytes (Figure 12).

### 2.13. GSK′872 Pretreatment Inhibited CaMKII Activation in Cardiomyocytes

**Figure 13:**
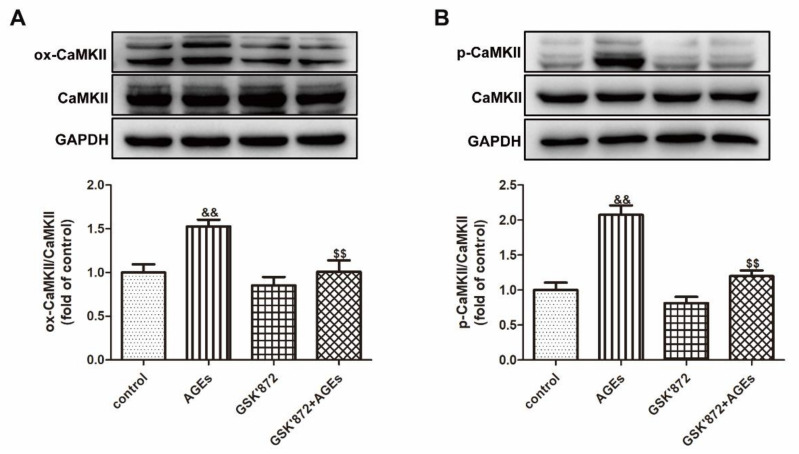
GSK′872 pretreatment inhibited CaMKII activation in cardiomyocytes. Cardiomyocytes were pretreated with 10 μM GSK′872 for 4 h followed by 200 μg/mL AGEs stimulation for 24 h. (**A**) The expression of ox-CaMKII and CaMKII was detected by Western blot in cardiomyocytes. GAPDH was used as a loading control. (**B**) The expression of p-CaMKII and CaMKII was detected by Western blot in cardiomyocytes. GAPDH was used as a loading control. Plots represent the mean ± SEM; n = 6. Statistical significance: ^&&^ *p* < 0.01 compared with control; ^$$^ *p* < 0.01 compared with AGEs.

Cardiomyocytes were pretreated with 10 μM GSK′872 for 4 h followed by 200 μg/mL AGEs stimulation for 24 h and detected the expression of ox-CaMKII and p-CaMKII. The study found that GSK′872 pretreatment significantly decreased the expression of ox-CaMKII and p-CaMKII induced by AGEs (Figure 13), suggesting that the GSK′872 attenuated CaMKII activation induced by AGEs.

### 2.14. GSK′872 Pretreatment Attenuated Oxidative Stress in Cardiomyocytes

**Figure 14:**
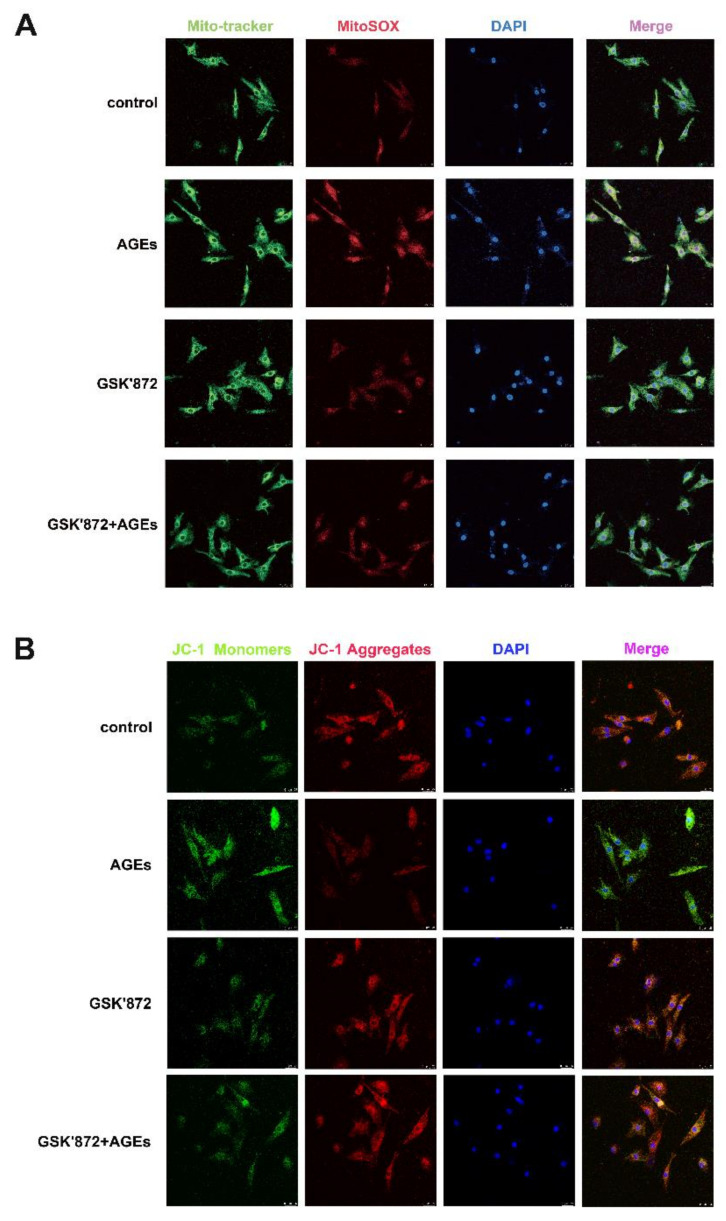
GSK′872 pretreatment attenuated oxidative stress in cardiomyocytes. Cardiomyocytes were pretreated with 10 μM GSK′872 for 4 h followed by 200 μg/mL AGEs stimulation for 24 h. (**A**) The generation of superoxide anion reactive oxygen species in mitochondria was detected by MitoSOX (Red) staining. Mito-Tracker (Green) co-localized mitochondria. The nuclei were stained by DAPI (Blue). Bar = 25 μm. (**B**) Mitochondrial membrane potential was detected by JC-1staining. When it is high, JC-1 aggregates in the matrix (Red). When it is low, JC-1 is mainly monomers (Green). The nuclei were stained by DAPI (Blue). Bar = 25 μm.

Cardiomyocytes were pretreated with 10 μM GSK′872 for 4 h followed by 200 μg/mL AGEs stimulation for 24 h. MitoSOX and JC-1 staining showed that GSK′872 pretreatment decreased the production of mitochondrial ROS (Figure 14A), improved the mitochondrial membrane potential (Figure 14B), suggesting that the GSK′872 pretreatment reduced oxidative stress induced by AGEs.

## 3. Discussion

DCM refers to abnormal cardiac structure and function in the absence of other cardiac risk factors. Recently, many intracellular pathways involved in the pathogenesis of DCM and potential strategies to protect DCM have been discovered, including antifibrotic agents, anti-inflammatory agents, and antioxidants. However, effective methods for preventing and treating DCM are still limited.

AGEs play a key role in the pathogenesis of cardiovascular diseases (CVDs) [32]. Previous studies have shown that whether you have diabetes or not, the high level of serum AGEs is related to the increased incidence of CVDs, such as arterial stiffness, atherosclerotic plaque formation, and endothelial dysfunction [33]. In diabetes, the high levels of glucose residues and metabolites promote AGEs production and destroy the function of cardiomyocytes and endothelial cells [34]. Based on the above research, this experiment used 200 μg/mL AGEs to stimulate cardiomyocytes for 24 h. It was found that after AGEs stimulation, the LDH release increased, but the ATP level decreased, which successfully simulated the cardiomyocyte damage in diabetes.

RIPK3 is an intracellular signaling protein that plays a key role in necroptosis. Studies have found that oxidized low-density lipoprotein upregulates the expression of RIPK3 and induces macrophages necroptosis in a RIPK3-dependent manner, leading to the atherosclerotic plaque formation [35,36]. A significant increase in the expression of RIPK3 mRNA and its phosphorylated protein can also be observed after myocardial infarction in rodents [37]. In addition, apoptosis is also an important manifestation of necroptosis. It should be noted that the activation of caspases mediates signal transduction and the execution of apoptotic cell death, while necroptosis can be activated under the condition of insufficient apoptosis, leading to cell death [38]. Our study found that AGEs increased the expression of RIPK3 and Cleaved-caspase 3, enhanced the ratio of TUNEL positive cardiomyocytes, suggesting that AGEs induced necroptosis in cardiomyocytes, which may be the important pathophysiological basis of cell damage.

CaMKII is a myocyte regulator associated with calcium homeostasis, which mediates cardiomyocyte death and cardiovascular related diseases [39,40]. CaMKII can be alternatively spliced. CaMKII δA is necessary for enhancing the L-type calcium current in neonatal cardiomyocytes [41]. During cardiac development, the CaMKII δA variant gradually transformed into the CaMKII δB and CaMKII δC variants [31]. CaMKII δB inhibits cardiomyocyte apoptosis induced by adriamycin and oxidative stress, and mediates cardioprotection [42,43]. In addition, CaMKII δC overexpression in mice accelerates the progression of heart failure and death [44]. We found that AGEs aggravated the disorder of CaMKIIδ alternative splicing, in which the mRNA expression of CaMKII δA and CaMKII δB decreased, but the mRNA expression of CaMKII δC increased.

Although CaMKII δB and CaMKII δC may have different or even opposite effects on cardiomyocyte viability, most studies have shown that excessive activation of CaMKII δ is involved in arrhythmia [45], interstitial fibrosis [46], and apoptosis [47]. The activation of CaMKII is enhanced in myocardial tissue of hypertrophic cardiomyopathy mice; inhibition of CaMKII activation improves diastolic function and delays the progression of cardiac remodeling [48]. In I/R injury, inhibiting CaMKII activation can reduce inflammation and fibrosis by regulating NF-κB and MAPK signaling pathways [27]. We found that AGEs increased the expression of ox-CaMKII and p-CaMKII and promoted CaMKII activation in cardiomyocytes.

In ischemia and oxidative stress injury, RIPK3 mediates the activation of CaMKII δ, induces myocardial necroptosis and apoptosis, and causes decompensated cardiac remodeling and heart failure [23]. However, it is not clear whether the AGEs-induced necroptosis of cardiomyocytes depends on the RIPK3-CaMKII pathway. Therefore, we downregulated the expression of RIPK3 by RIPK3 siRNA transfection, and found that RIPK3 downregulation corrected CaMKII δ alternative splicing disorder, inhibited CaMKII activation, reduced oxidative stress, attenuated necroptosis, and improved cell damage in cardiomyocytes.

The above studies have shown that RIPK3 downregulation has a protective effect on the heart, suggesting that RIPK3 is a potential target to reduce AGEs-induced cardiomyocyte damage. Pharmacological reduction of RIPK3 expression may provide a new strategy for the prevention and treatment of DCM.

The RIPKs family contains seven members, RIPK1-7, which are key mediators of cell survival, cell death, and death domain receptor signal transduction, cell stress, and inflammation [49]. At present, many small molecule drugs targeting RIPK1 have been successfully developed. Among them, the first RIPK1 inhibitor GSK2982772 has entered clinical trials for the treatment of ulcerative colitis, psoriasis, rheumatoid arthritis, and other inflammatory diseases [50]. Based on this, the research of small molecule RIPK3 inhibitors has also received more and more support. Compared with RIPK1 inhibitors, RIPK3 inhibitors can protect cells from more extensive harmful stimulation. RIPKs inhibitors have three kinase binding modes. Type I kinase inhibitors occupy the ATP binding site and target the active DFG-in conformation [51]. Type II kinase inhibitors target the ATP binding site and combine with the inactive DFG-out state [52]. Type III kinase inhibitors bind to the hydrophobic pocket of the kinase domain and target the inactive DFG-out state, with or without ATP competition. GSK′872 is a small molecule inhibitor with high affinity to the kinase domain of RIPK3 and specifically inhibits RIPK3 activity. However, the specific binding mode of RIPK3 is not yet clear, so it is an unclassified inhibitor. A preclinical study has proved that GSK′872 can reduce ischemic brain injury [21]. Similarly, in the middle cerebral artery embolism, GSK′872 significantly reduced cerebral infarction volume [53]. However, the role of GSK′872 in the prevention and treatment of DCM remains unclear. We pretreated cardiomyocytes with GSK′872 for 4 h and then stimulated with AGEs for 24 h. The results showed that RIPK3 inhibitor GSK′872 corrected CaMKIIδ alternative splicing disorder, inhibited CaMKII activation, reduced oxidative stress, attenuated necroptosis, and improved cell damage in cardiomyocytes. GSK′872 may be a potential small molecule drug for the prevention and treatment of diabetic cardiomyopathy.

However, our research still has some limitations. This study mainly clarified the upstream and downstream relationship between RIPK3, CaMKII, and necroptosis by RIPK3 downregulation. It lacked the verification of the regulation feedback test of CaMKII and necroptosis. In addition, there was no overall animal-level discussion.

**Figure 15:**
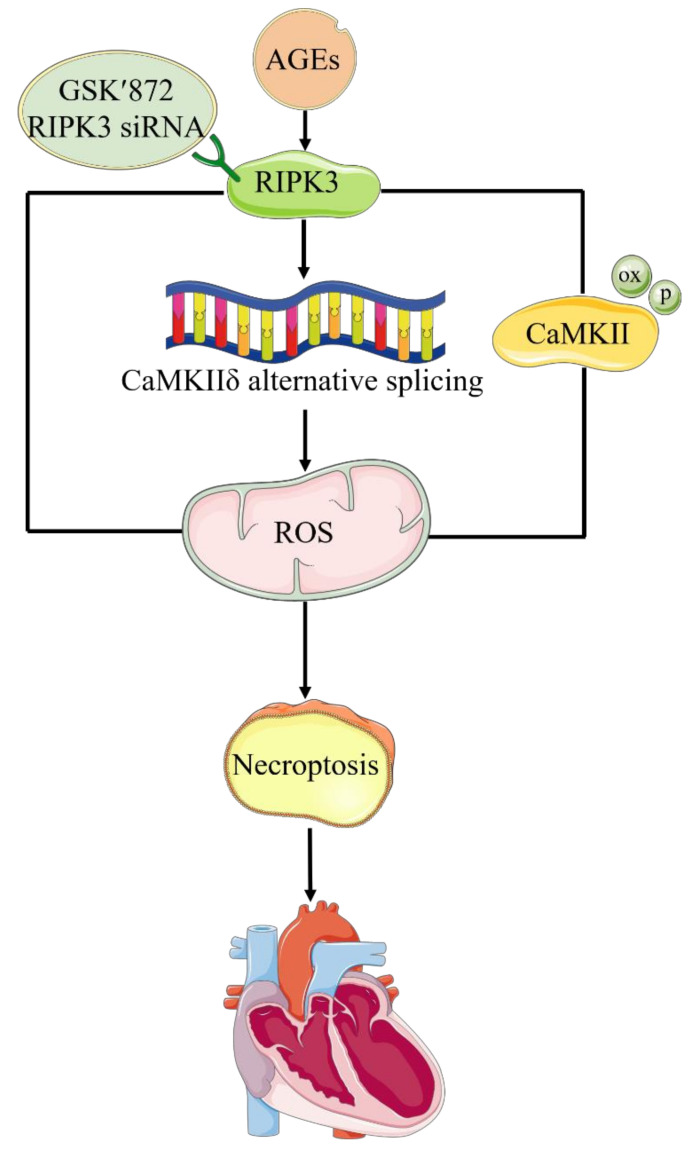
Mechanism of AGEs-induced necroptosis in cardiomyocytes. AGEs increased the expression of RIPK3, aggravated the disorder of CaMKII δ alternative splicing, promoted CaMKII activation, enhanced oxidative stress, induced necroptosis, and damaged cardiomyocytes. RIPK3 downregulation or RIPK3 inhibitor GSK′872 corrected CaMKII δ alternative splicing disorder, inhibited CaMKII activation, reduced oxidative stress, attenuated necroptosis, and improved cell damage in cardiomyocytes.

In conclusion, AGEs increased the expression of RIPK3, aggravated the disorder of CaMKII δ alternative splicing, promoted CaMKII activation, enhanced oxidative stress, induced necroptosis, and damaged cardiomyocytes. RIPK3 downregulation or RIPK3 inhibitor GSK′872 corrected CaMKII δ alternative splicing disorder, inhibited CaMKII activation, reduced oxidative stress, attenuated necroptosis, and improved cell damage in cardiomyocytes (Figure 15). The research suggests that RIPK3 may be an important target of diabetic myocardial damage, which provides a new idea for prevention and treatment of diabetic cardiomyopathy.

## 4. Methods and Materials

### 4.1. Cell Culture and Treatment

The primary cardiomyocytes were isolated from 1–3-day-old Sprague Dawley (SD) rats by trypsin (Beyotime, Shanghai, China) digestion. The cardiomyocytes were cultured in DME/F-12 medium (Hy Clone, Logan, UT, USA) containing 10% FBS (Gibco, Temecula, CA, USA) for 24 h. The cardiomyocytes were subsequently treated with AGEs (200 μg/mL, BioVision, Milpitas, CA, USA) for 24 h. In addition, cardiomyocytes were transfected with RIPK3 siRNA (RiboBio, Guangzhou, China) or RIPK3 inhibitor GSK′872 (10 μM, GlpBio, Montclair, CA, USA) for pretreatment for 4 h followed by AGEs stimulation for 24 h.

The experiment complied with Guidelines for Care and Use of Laboratory Animals from the US National Institutes and was approved by the Instructional Animal Care and Use Committee of Nantong University (approval no. NTU-20161225).

### 4.2. RIPK3 siRNA Transfection

Three RIPK3 siRNA (001: 5′-GGAAAGGCTTCTAAAGCAA-3′, 002: 5′-GCTGGAGTTCTGAGCCTAA-3′, 003: 5′-GGACTGAAGGAGTTAATGA-3′) and NC siRNA (RiboBio, Guangzhou, China) were obtained commercially.

After serum deprivation for 24 h, cardiomyocytes were transfected with the above-mentioned siRNA into cardiomyocytes by lipofectamine 2000 (Invitrogen, Carlsbad, CA, USA) for 4 h followed by AGEs stimulation for 24 h.

### 4.3. Lactate Dehydrogenase (LDH) Measurement

LDH release into the culture medium was detected by LDH Cytotoxicity Assay Kit (Beyotime, Shanghai, China) according to the manufacturer’s instructions. Cardiomyocytes were cultured in a 96-well plate with density less than 80–90%. After treatment, 120 μL supernatant of each well and 60 μL LDH detection working solution were mixed and incubated for 30 min in the dark. LDH release into the culture medium was calculated according to the absorbance at 490 nm.

### 4.4. Adenosine Triphosphate (ATP) Measurement

Cellular ATP level was detected by CellTiter-Lumi™ Luminescent Cell Viability Assay Kit (Beyotime, Shanghai, China) according to the manufacturer’s instructions. About 20,000 cardiomyocytes were cultured in each well of a 96-well plate. After equilibrating to room temperature, 100 µL ATP detection reagent was mixed with culture medium in each well, and incubated in the dark for 10 min. The ATP content of cardiomyocytes was calculated according to chemiluminescence reading.

### 4.5. Real-Time PCR

Cardiomyocytes were lysed with Trizol to obtain total RNA. RNA samples of cardiomyocytes were reverse transcribed into cDNA according to Prime Script™ RT Master Mix Kit (Takara, Kyoto, Japan) instructions. CaMKIIδ and housekeeper mRNA primer sequences were synthesized by Sangon Biotech (Shanghai, China) (Table 1). Each group of cardiomyocyte cDNA was analyzed by real-time PCR three times. 18S was used as the reference gene group. The relative expression level of CaMKIIδA, CaMKIIδB, and CaMKIIδC mRNA was calculated according to the reference.

### 4.6. Western Blot

Cardiomyocyte protein was extracted by NP-40 Lysis Buffer (Beyotime, Shanghai, China) and PMSF (Beyotime, Shanghai, China). The protein samples were separated by sodium dodecyl sulfate-polyacrylamide gel electrophoresis (SDS-PAGE) and transferred to polyvinylidene fluoride (PVDF) membrane (Millipore, Burlington, MA, USA). After being blocked by 5% skim milk for 2 h, the PVDF membrane was incubated with the primary antibody at 4 °C overnight. The primary antibody solution included: anti-RIPK3 (1:1000, Novusbio, Littleton, CO, USA), anti-caspase 3, anti-Cleaved-caspase3 (1:1000, Cell Signaling Technology, Danvers, MA, USA), anti-CaMKII (1:1000, Abcam, Cambridge, UK), anti-ox-CaMKII (1:1000, Millipore, NJ, USA), anti-p-CaMKII (1:1000, Thermo Fisher Scientific, Rockford, IL, USA), anti-GAPDH (1: 5000, CONSON, Shanghai, China). Then, the PVDF membrane was incubated with horseradish peroxidase (HRP)-conjugated IgG (ZSGB-BIO, Beijing, China) for 2 h. The enhanced chemiluminescence solution (ECL, Thermo Fisher Scientific Inc., Rockford, IL, USA) was dropped on the PVDF membrane to observe the protein band.

### 4.7. Immunofluorescence Staining

Cardiomyocytes were cultured in cell climbing sheets. After treatment, cardiomyocytes were incubated with anti-RIPK3 (1:100, Novusbio, Littleton, CO, USA) at 4 °C overnight. Next, cardiomyocytes were incubated with Alexa Fluor 488 (1:500, Beyotime, Shanghai, China) for 2 h. RIPK3 expression was evaluated according to the fluorescence intensity.

### 4.8. TdT-Mediated dUTP Nick End Labeling (TUNEL) Staining

The apoptosis of cardiomyocytes was detected by One Step TUNEL Apoptosis Assay Kit (Beyotime, Shanghai, China) according to the manufacturer’s instructions. Cardiomyocytes were cultured in cell climbing sheets. After treatment, cardiomyocytes were incubated with 50 μL TUNEL detection solution at 37 °C for 1 h in the dark. The apoptosis of cardiomyocytes was assessed according to the green fluorescence intensity.

### 4.9. MitoSOX and Mito-Tracker Staining

Cardiomyocytes were cultured in cell climbing sheets. After treatment, cardiomyocytes were incubated with MitoSOX Red (YEASEN, Shanghai, China) and Mito-Tracker Green (Beyotime, Shanghai, China) staining solutions at 37 °C for 15 min in the dark. The mitochondrial oxidative stress was evaluated according to fluorescence intensity.

### 4.10. Mitochondrial Membrane Potential (ΔΨm) Measurement

The mitochondrial membrane potential of cardiomyocytes was detected by Mitochondrial Membrane Potential Assay Kit with JC-1 (Beyotime, Shanghai, China) according to the manufacturer’s instructions. Cardiomyocytes were cultured in cell climbing sheets. After treatment, cardiomyocytes were incubated with JC-1 staining solution at 37 °C for 20 min in the dark. The ΔΨm was evaluated according to the red fluorescence intensity of JC-1 aggregates and the green fluorescence intensity of JC-1 monomers.

### 4.11. Statistical Analysis

The data were expressed as mean ± standard error (±S.E.M), processed by GraphPad Prism software, and analyzed by one-way ANOVA, Student–Newman–Keuls (SNK) test. *P*-values lower than 0.05 were considered statistically significant.

## Figures and Tables

**Table 1:** The sequences of the primers for real-time PCR.

Gene	Forward Primer	Reverse Primer
CaMKⅡδA	5′-CGAGAAATTTTTCAGCAGCC-3′	5′-ACAGTAGTTTGGGGCTCCAG-3′
CaMKⅡδB	5′-CGAGAAATTTTTCAGCAGCC-3′	5′-GCTCTCAGTTGACTCCATCATC-3′
CaMKⅡδC	5′-CGAGAAATTTTTCAGCAGCC-3′	5′-CTCAGTTGACTCCTTTACCCC-3′
18S	5′-AGTCCCTGCCCTTTGTACACA-3′	5′-CGATCCGAGGGCCTCACTA-3′

## Data Availability

Not applicable.

## Abbreviations

DCM	Diabetic cardiomyopathy
DM	Diabetes mellitus
AGEs	Glycation end products
RIPK3	Receptor interacting proteins kinase 3
CaMKII	Calcium/calmodulin-dependent protein kinase II
Thr287	Threonine-287
Met281/282	Methionine-281/282
mPTP	Mitochondrial permeability transition pore
ΔΨm	Mitochondrial membrane potential
SD	Sprague Dawley
